# Long-Range Coulomb Effect in Intense Laser-Driven Photoelectron Dynamics

**DOI:** 10.1038/srep27108

**Published:** 2016-06-03

**Authors:** Wei Quan, XiaoLei Hao, YongJu Chen, ShaoGang Yu, SongPo Xu, YanLan Wang, RenPing Sun, XuanYang Lai, ChengYin Wu, QiHuang Gong, XianTu He, XiaoJun Liu, Jing Chen

**Affiliations:** 1 State Key Laboratory of Magnetic Resonance and Atomic and Molecular Physics and Center for Cold Atom Physics, Wuhan Institute of Physics and Mathematics, Chinese Academy of Sciences, Wuhan 430071, China; 2 Institute of Theoretical Physics and Department of Physics, Shanxi University, 030006 Taiyuan, China; 3 School of Physics, University of Chinese Academy of Sciences, Beijing 100080, China; 4 State Key Laboratory for Mesoscopic Physics, Department of Physics, Peking University, Beijing 100871, China; 5 Collaborative Innovation Center of Quantum Matter, Beijing, China; 6 HEDPS, Center for Applied Physics and Technology, Collaborative Innovation Center of IFSA, Peking University, Beijing 100084, China; 7 Institute of Applied Physics and Computational Mathematics, P. O. Box 8009, Beijing 100088, China

## Abstract

In strong field atomic physics community, long-range Coulomb interaction has for a long time been overlooked and its significant role in intense laser-driven photoelectron dynamics eluded experimental observations. Here we report an experimental investigation of the effect of long-range Coulomb potential on the dynamics of near-zero-momentum photoelectrons produced in photo-ionization process of noble gas atoms in intense midinfrared laser pulses. By exploring the dependence of photoelectron distributions near zero momentum on laser intensity and wavelength, we unambiguously demonstrate that the long-range tail of the Coulomb potential (i.e., up to several hundreds atomic units) plays an important role in determining the photoelectron dynamics after the pulse ends.

In atomic physics, the Coulomb interaction no doubt plays an essential role. Its long range characteristic is indispensable for our understanding on structure and dynamics of atoms. For example, the dynamics of the highly excited Rydberg atom in external static electric or magnetic fields is determined by the interplay of the intrinsic ionic long-range Coulomb field with external fields^1^. On the other hand, the role of ionic Coulomb potential has for a long time been ignored in the dynamics of atoms subject to ultrashort intense laser fields. Therein, an intuitive simplified simple-man model^2^,^3^, in which the Coulomb potential is completely ignored, has been applied to comprehend the underlying mechanisms of various highly nonlinear phenomena observed in atom-intense laser interactions and achieved astonishing successes in descriptions of, e.g., the above-threshold ionization (ATI), high harmonic generation and non-sequential double ionization (NSDI) etc.^2^,^3^,^4^,^5^,^6^,^7^,^8^,^9^,^10^.

In fact, the effect of the Coulomb potential of the ionic core, e.g. Coulomb focusing effect, has been investigated in above-threshold ionization^11^,^12^,^13^ and non-sequential double ionization^14^,^15^ years ago. In the ATI process, a cusp-like structure has been identified experimentally in the transverse momentum distributions for noble gas atoms subject to intense laser field^12^,^13^. The physical origin of this cusp-like structure is in accordance with the “target cusp” in the electron momentum distributions observed in the experiment on the collision of ions^16^,^17^, which is believed to be due to the long-range Coulomb interaction between the photoelectron and the remaining ion. Recently, the Coulomb potential effect has attracted an increasing attention since low-energy structure (LES) was experimentally observed in above-threshold ionization spectrum^18^,^19^,^20^. It has been commonly accepted that the Coulomb potential of the parent ionic core is responsible for the LES^18^,^19^,^20^,^21^,^22^,^23^,^24^,^25^. Although, in principle, the Coulomb potential (~ −1/*r* (in atomic units (a.u.))) extends to infinity, a relatively short range of the ionic potential (i.e., from several tens to about 100 atomic units) has been identified to be necessary to generate the observed LES (including high low-energy structure (HLES) and very low-energy structure (VLES))^24^,^25^. Specifically, Coulomb focusing, which means that the attractive Coulomb potential will lead to concentration of the photoelectrons in the longitudinal momentum distributions, is believed to be responsible for the formation of the HLES (energy part above ~1 eV in the LES)^18^,^19^,^21^,^22^. On the other hand, the VLES (energy part below ~1 eV) observed in the photoelectron spectrum is found to be connected with double-hump structure (DHS) in the momentum distributions^11^,^20^,^26^ and a bunching effect has been proposed to be its underlying mechanism^20^,^24^. In this case, the Coulomb attraction is only significant when the photoelectron moves close to the ionic core (the distance is much less than the quiver amplitude (*α* = *E*/*ω*^2^) of the electron in the laser field, which is about 100 a.u. for a typical laser field with *I* = 10^14^ W/cm^2^ and *λ* = 2000 nm) and the energy bunching may occur even in a potential with a range of about several tens atomic units^24^.

In contrast to recent accumulating experimental facts that, in general, the Coulomb potential plays a pivotal role in the photo-ionized electron dynamics, however, it is still unclear to which extent the Coulomb potential, and especially *its long range tail*, which can in principle extends up to infinity, would determine the photoelectron dynamics. On the other side, from physical point of view, the effect of the long-range Coulomb potential tail is expected to be rather faint and more importantly, is always inevitably entangled with much stronger effects from the inner part of the Coulomb potential as well as the strong external field. Therefore, to uncover the effect of extremely long range (e.g., up to several hundreds a.u.) Coulomb potential tail, one has to perform precision measurements with a delicate procedure and most importantly, to extract it from the dominant influence of the inner part of the Coulomb potential and the intense laser field, which poses a tremendous challenge for both experimental and theoretical studies. It is noteworthy that which range of the potential corresponds to the cusp structure in refs 12, 13, 16 and 17 has also not been identified.

In this work, we overcome this hurdle by disentangling the effect of the long range Coulomb potential tail from the dominant effects related to the inner part of Coulomb potential and the external field by investigating the evolution of photoelectron momentum distribution very close to zero with respect to laser intensities and wavelengths. For our case, the long range Coulomb potential tail solely plays a significant role in the dynamics of the near-zero momentum photoelectrons, which have moved far away from the parent ionic core when the laser field ends. We illustrate this by plotting in Fig. 1 the calculated spatial distributions of photoelectrons generated in the laser field with wavelength of 1300 nm and 2400 nm and intensities of 2.0 and 3.0 × 10^13^ W/cm^2^, respectively, right after the laser pulses end. One finds in Fig. 1 that some of the photoelectrons may move to a distance of several hundreds of a.u. from the parent ionic core right after the laser pulse vanishes. Although quite far away from the core, the photoelectrons are still subject to the long range tail of the Coulomb field. After decelerated by the Coulomb potential tail, the photoelectrons will be finally measured by the detector, which can be considered to be infinitely far away from the ionic core. As we will show below, those electrons will eventually form a peak structure locating near momentum of *p* = 0 in the final photoelectron momentum distribution. These near-zero momentum electrons measured in our experiments can be taken as a probe of the effect of the long-range Coulomb potential tail. Moreover, as also shown in Fig. 1, the photoelectron spatial distributions right after the end of the laser pulse can be controlled by laser wavelength and intensity. A higher laser intensity or a longer wavelength results in a broader spatial distribution under otherwise identical conditions. Consequentially, the final photoelectron momentum distribution will vary with laser parameters. The effect of the long range Coulomb potential tail can thus be unraveled by comparing the photoelectron momentum distributions under different laser parameters, i.e., laser wavelength and intensity.

## Results

We perform the measurements with cold target recoil-ion momentum spectroscopy (COLTRIMS)^27^,^28^. Laser beam at midinfrared wavelength is generated with a commercial optical parametric amplifier (OPA) pumped by a Ti: Sapphire laser system. The midinfrared laser pulse is focused into the cold supersonic beam inside the vacuum chamber of COLTRIMS, and the 3 dimensional momenta of photoelectrons and photoions are measured coincidently. A detailed description of our experimental procedure can be found in Methods.

**Figure 2:**
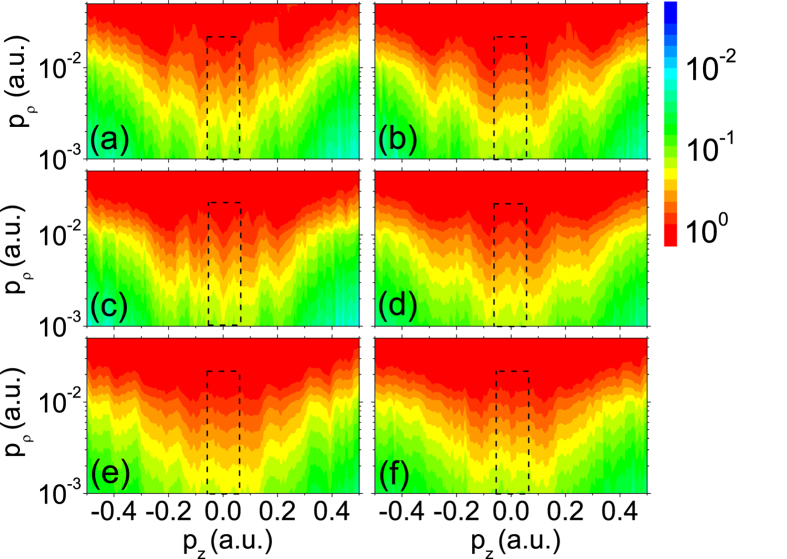
Measured two dimensional photoelectron momentum distributions for Xe. The laser field is linearly polarized in z direction. (**a**) *I* = 2.0 × 10^13^ W/cm^2^, *λ* = 2400 nm; (**b**) *I* = 3.0 × 10^13^ W/cm^2^, *λ* = 2400 nm; (**c**) *I* = 2.1 × 10^13^ W/cm^2^, *λ* = 1800 nm; (**d**) *I* = 2.9 × 10^13^ W/cm^2^, *λ* = 1800 nm; (**e**) *I* = 2.7 × 10^13^ W/cm^2^, *λ* = 1300 nm; (**f**) *I* = 5.1 × 10^13^ W/cm^2^, *λ* = 1300 nm. The dashed rectangle highlights the ZES.

The typical two dimensional photoelectron momentum distributions (PMDs) with respect to laser wavelength and intensity are shown in Fig. 2. The laser field is linearly polarized along z direction. The distributions are confined to 0.001 a.u. < *p*_*ρ*_ < 0.05 a.u. in log scale, where 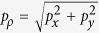, and −0.5 a.u. < *p*_*z*_ < 0.5 a.u. in linear scale. Note that the PMDs are normalized by the yields at *p*_*z*_ = 0 and *p*_*ρ*_ = 0.02 a.u. for visual convenience. Due to low statistics in this extremely low momentum region, the data have been smoothed to better resolve the fine structures in this area. Two series of peaks in both positive and negative z directions can be distinguished in all PMDs. These peaks correspond to the LES and VLES or high-order LESs^20^,^24^,^29^,^30^, which are not the focus here. More interestingly, it can be seen that a peak structure at *p*_*z*_ = 0 and *p*_*ρ*_ < 0.02 a.u., which is highlighted with a dashed black rectangle in each panel, manifests in all spectra irrespective of the laser conditions. Dependence of its amplitude versus laser intensity and wavelength is also visible upon careful inspection. Because this central peak structure exhibits identical *p*_*ρ*_ and *p*_*z*_ momentum intervals to those of the zero-energy-structure (ZES), which has been reported for the first time by Dura *et al.*^31^ for Ar and O_2_ in a laser field with a much longer wavelength of 3100 nm, we dub it ZES too. It is suggested that the ZES is attributed to ionization of the Rydberg atoms by the extraction field of COLTRIMS^29^. However, our measurements (not shown here) with extraction fields of 2.3 V/cm and 7.4 V/cm hardly show any difference, implying that the mechanism of field ionization of the Rydberg atoms cannot be applied to explain our experimental results. Therefore, the underlying mechanism of the ZES has not been well understood.

### The semiclassical model

To understand the underlying physics of ZES, we study the ionization dynamics using a semiclassical model (for details, see Methods). Our calculation procedure is based on a well-verified numerical method which has been employed successfully to provide physical insight into intense field atomic ionization processes such as ATI and NSDI, see e.g.^11^,^19^,^20^,^32^,^33^. In our calculation, the bound electron is firstly tunnel ionized in the combined laser electric field and atomic Coulomb field. The initial tunnel exit, the initial transverse velocity and the weight of the electron trajectory are calculated according to the tunneling model^32^,^34^. After ionization, the evolution of the free electron is governed by the 3 dimensional Newton’s equations of motion and the Coulomb interaction between electron and parent ionic core is fully considered. After the laser pulse vanishes, the momentum of electron eventually registered by the detector (*t* → ∞) can be obtained analytically (for details, see Methods).

### Comparison of measured and calculated momentum distributions

**Figure 3:**
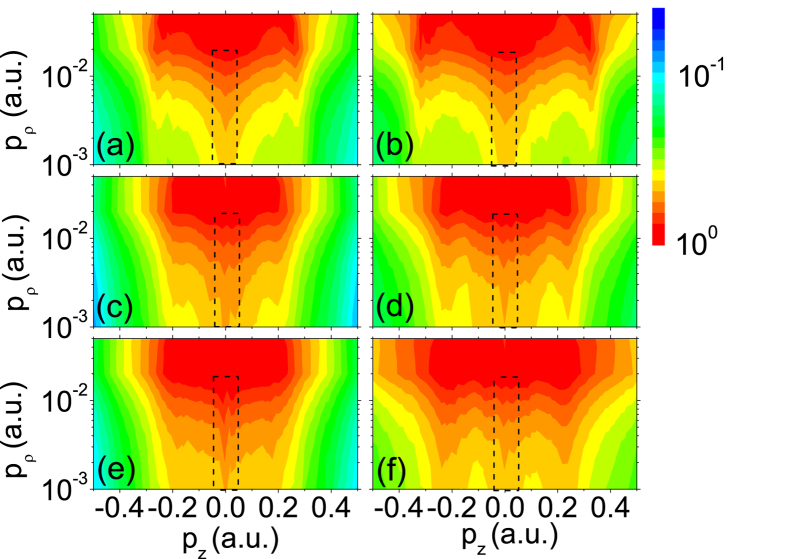
Calculated two dimensional photoelectron momentum distributions for Xe. The parameters are chosen according to the experiments. (**a**) *I* = 2.0 × 10^13^ W/cm^2^, *λ* = 2400 nm; (**b**) *I* = 3.0 × 10^13^ W/cm^2^, *λ* = 2400 nm; (**c**) *I* = 2.1 × 10^13^ W/cm^2^, *λ* = 1800 nm; (**d**) *I* = 2.9 × 10^13^ W/cm^2^, *λ* = 1800 nm; (**e**) *I* = 2.7 × 10^13^ W/cm^2^, *λ* = 1300 nm; (**f**) *I* = 5.1 × 10^13^ W/cm^2^, *λ* = 1300 nm. The dashed rectangle highlights the ZES.

The calculated PMDs are shown in Fig. 3 for Xe with identical laser parameters to those in Fig. 2. The main features of the experimental results in Fig. 2 are well reproduced in the semiclassical simulations: the LES peaks and a central peak structure at *p*_*z*_ = 0, which is highlighted with a dashed black rectangle in each panel, appears in all the PMDs, and moveover, the ZES peak amplitude with respect to the LES varies with laser intensity and wavelength.

**Figure 4:**
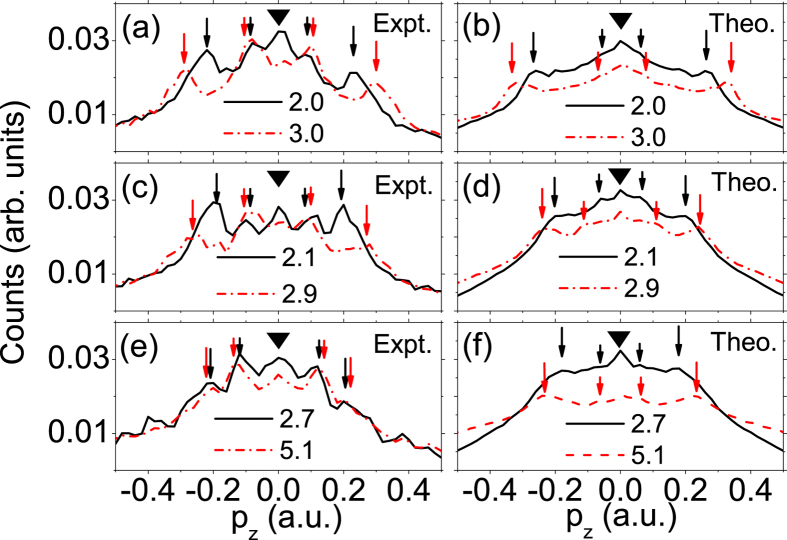
The measured (**a,c,e**) and calculated (**b,d,f**) longitudinal momentum distributions (LMDs) with integration over a small interval (0–0.02 a.u.) of the transverse momentum. The laser intensity is labelled in the unit of 10^13^ W/cm^2^. (**a,b**) *λ* = 2400 nm; (**c,d**) *λ* = 1800 nm; (**e,f**) *λ* = 1300 nm. The inverted triangle, short and long vertical arrows indicate the ZES, VLES or high-order LES and LES, respectively, in each panel.

To compare the experimental data and the calculation results more carefully, the measured and calculated longitudinal momentum distributions (LMDs) integrated within the transverse momentum interval of (0, 0.02 a.u.) are shown in Fig. 4. The total yield of each curve has been normalized. At all three wavelengths, i.e., 2400 nm (panel (a)), 1800 nm (panel (c)) and 1300 nm (panel (e)), there is a peak at *p*_*z*_ = 0, which corresponds to the ZES observed in Fig. 2 and is highlighted with an inverted black triangle in each panel. Besides this peak, in all three panels, there are two or three pairs of peaks at nonzero momentum distributed symmetrically with respect to *p*_*z*_ = 0. These peaks can be attributed to the LES peaks and their momenta are in good agreement with previous theoretical predictions^24^,^30^. The position of the lowest order LES is indicated with a long vertical arrow and the VLES or high-order LES a short vertical arrow in each panel. Upon a closer inspection, it is also found that the ZES peak at *p*_*z*_ = 0 shrinks with respect to the LES when the laser intensity is increased. At the same time, the momenta of the LES peaks increase noticeably with respect to the rising intensity. The different intensity dependence for the ZES and LES might suggest a different physical origin for them. In panels (b), (d) and (f), where the semiclassical calculation results are presented, all the main features in the experimental data are reproduced, i.e., the LES peaks shift with increasing intensity and the amplitude of the ZES is suppressed with respect to the LES for higher intensity.

## Discussion

**Figure 5:**
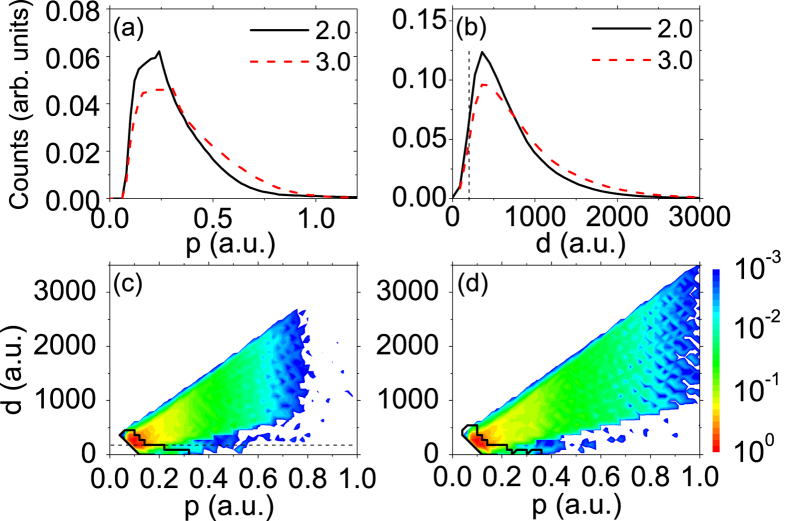
Calculated photoelectron distributions right after the laser pulse ends. The laser wavelength is 2400 nm and the laser intensity is labelled in the unit of 10^13^ W/cm^2^. (**a**) Photoelectron yields versus the total momentum, *p*. (**b**) Photoelectron yields versus its distance from the ionic core, *d*. (**c,d**) Photoelectron momentum and spatial distributions (MSDs) for 2.0 × 10^13^ W/cm^2^ and 3.0 × 10^13^ W/cm^2^, respectively. The dashed straight lines in (**b,c**) indicate the edge of the truncated model potential (See Fig. 7(c,d) and related text). Photoelectrons inside the areas circled by black curves in (**c,d**) correspond to those that will eventually form ZES at *t* = ∞. See text for details.

In order to further facilitate the analysis, we present in Fig. 5 the calculated photoelectron momentum distributions and spatial distributions right after the laser pulse ends, i.e., at *t*_1_ = *τ*_*p*_ + 3 × *T*, for laser wavelength of 2400 nm. In Fig. 5(a), we depict the photoelectron yields with respect to the sum momentum, 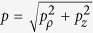, at *t*_1_. There is a striking dip around *p* = 0 because the electrons with negative total energy, i.e., the sum of their kinetic energy and Coulomb potential is less than zero, are removed. Figure 5(a) shows that more photoelectrons appear in the low momentum region for low intensity than for high intensity. In Fig. 5(b), we depict photoelectron distributions with respect to its distance from the parent ionic core (i.e., 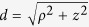) right after the laser pulse ends (i.e., at *t*_1_). As shown, more photoelectrons distribute closer to the core at lower laser intensity. To shed more light on the physical origin of the ZES, we present in Fig. 5(c,d) the two dimensional photoelectron Momentum and Spatial Distributions (MSDs), i.e., the photoelectron distributions with respect to the total momentum, *p*, of the photoelectron and its distance from the ionic core, *d*, right after the laser pulse ends. For the photoelectrons in the areas circled by the black curves, they will be subject to the Coulomb potential strong enough to form the ZES eventually. From the shape of the areas we can find that the farthest electrons, which constitute the ZES, locate around 500 a.u. and possess momenta less than 0.1 a.u. at *t*_1_. On the other hand, the photoelectrons with momenta around 0.3 a.u. can also contribute to the ZES if they locate close enough to the core. Generally, for higher laser intensity, the photoelectron distribution covers a larger area, while the area for the ZES is almost identical, independent of laser intensity. It can be understood that, based on the above analysis, the photoelectrons locate closer to the ionic core and possess smaller momentum at *t*_1_ for lower intensity. As a consequence, they will feel relatively stronger Coulomb attraction and be easier to be influenced, resulting in a more pronounced ZES structure at lower intensity. The above analysis leads to the conclusion that the physical origin of the ZES is the deceleration effect of the long-range Coulomb potential on low momentum photoelectrons during their passage to the detector after the laser pulse ends.

**Figure 6:**
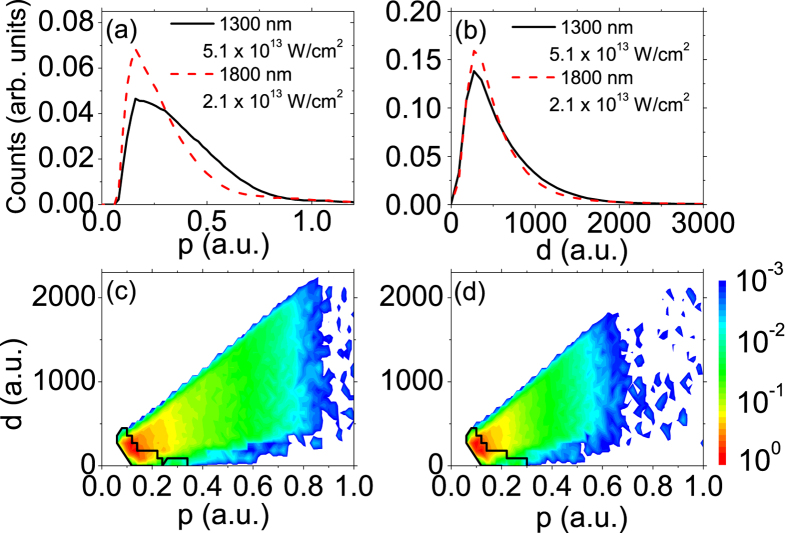
Calculated photoelectron distributions right after the laser pulse ends. (**a**) Photoelectron yields versus the total momentum, *p*. (**b**) Photoelectron yields versus its distance from the ionic core, *d*. (**c,d**) Photoelectron momentum and spatial distributions (MSDs) for *I* = 5.1 × 10^13^ W/cm^2^, *λ* = 1300 nm and *I* = 2.1 × 10^13^ W/cm^2^, *λ* = 1800 nm, respectively. Photoelectrons inside the areas circled by black curves in (**c,d**) correspond to those that will eventually form ZES at *t* = ∞. See text for details.

As the Coulomb interaction is exclusively determined by the distance between the photoelectron and the ionic core, we further compare the ZES of different wavelengthes and intensities but with equal quiver amplitudes. In the experiment, we set the laser intensities and wavelengths in two cases with close quiver amplitudes: One is at 1800 nm and 2.1 × 10^13^ W/cm^2^, for which the photoelectron quiver amplitude is 38.2 a.u. The other one is at 1300 nm and 5.1 × 10^13^ W/cm^2^, corresponding to a quiver amplitude of 31.1 a.u. From Fig. 4(c,e), one can see that, although the excursion distances are close for each other, the ZES is much more prominent for longer wavelength, which is also consistent with the semiclassical calculations presented in Fig. 4(d,f). To perceive the physics behind, we depict in Fig. 6 the calculated momentum and spatial distributions of photoelectrons for both cases at *t*_1_, i.e., right after the laser pulse ends. From Fig. 6(a), one can find that the two momentum distributions are significantly different and considerably more photoelectrons for longer wavelength possess lower momentum than for shorter wavelength. While Fig. 6(b) shows that slightly more photoelectrons locate in short distance region for longer wavelength than for shorter wavelength. This can be understood by the fact that the quiver amplitude is proportional to *E*/*ω*^2^, while the ponderomotive energy, which determines the kinetic energy of the photoelectron acquired from the laser field, is proportional to *E*^2^/*ω*^2^. Although the quiver amplitudes for two cases are close (the spatial distributions are similar, as shown in Fig. 6(b)), the ponderomotive energy is considerably smaller for the longer wavelength case. By comparison of Fig. 6(c,d), it can be found that the areas circled by the black curves (corresponding to the ZES) are almost identical for two cases, while the whole MSD distribution at *t*_1_ covers a larger area for shorter wavelength (Fig. 6(c)). Therefore, the result that the ZES is more significant for longer wavelength can be understood by a stronger long-range Coulomb potential effect in this case, as more photoelectrons appear closer to the ionic core and possess lower momenta. It is worthwhile mentioning that our result is consistent with the observation of Wolter *et al.*^35^ that the ZES of Xe is barely visible for longer wavelength (*λ* = 3100 nm) and relatively high intensity (*I* = 4.0 × 10^13^ W/cm^2^) which correspond to higher ponderomotive energy and larger quiver amplitude than those in our experiments.

**Figure 7:**
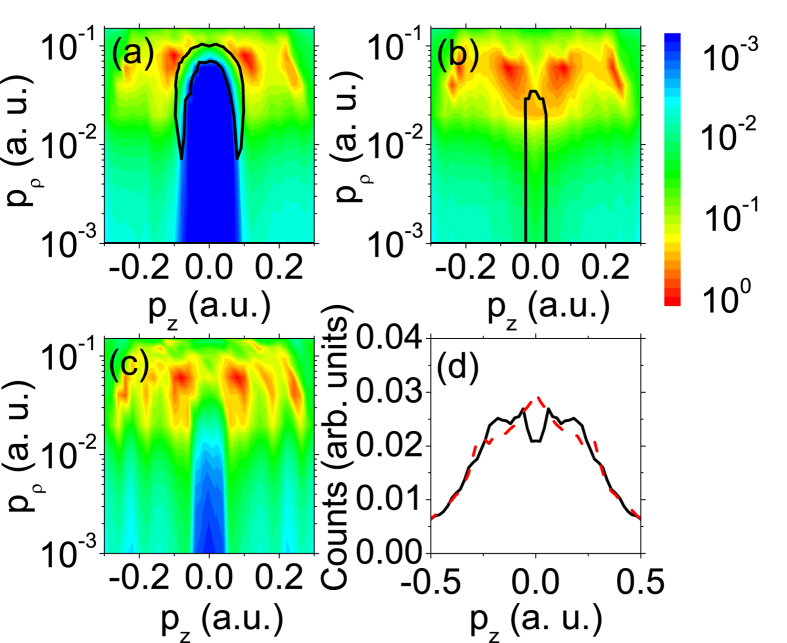
(**a,b**) Calculated two dimensional photoelectron momentum distributions of Xe for *t* = *t*_1_ and t = ∞, respectively. Electrons inside the area circled by the black curve in (**a,b**) correspond to those that will eventually form ZES structure. The laser wavelength is *λ* = 2400 nm and the laser intensity is *I* = 2.0 × 10^13^ W/cm^2^. (**c**) Calculated two dimensional photoelectron momentum distribution at *t* = ∞ with a truncated model potential instead of Coulomb potential. The parameters are identical to those of (**a**). (**d**) Calculated longitudinal momentum distribution integrated over a small interval (0–0.02 a.u.) of the transverse momentum at *t* = ∞ with a truncated model potential (black solid line) and Coulomb potential (red dashed line). The parameters are identical to those of (**c**). See text for details.

To illustrate the perceptible role of the long-range Coulomb potential, we present in Fig. 7(a,b) the calculated PMDs with laser wavelength of 2400 nm and intensity of 2.0 × 10^13^ W/cm^2^ at *t* = *t*_1_ (i.e., right after the laser pulse ends) and *t* = ∞ (i.e., when the photoelectrons hit the detector), respectively. The comparison of Fig. 7(a,b) shows how the ZES structure develops. It is noteworthy that the photoelectrons with total energy *E* < 0 are dropped in Fig. 7(a), so a hole centered at origin appears in the PMDs at *t* = *t*_1_. Additionally, the photoelectrons contributing to the ZES form a ring, as circled by the black curve beside the boundary of the hole. At *t*_1_, as shown in Fig. 5(b), these electrons are still subject to the Coulomb potential and their distances from the ionic core peak at about 300 a.u. With evolution of time, the momenta of the photoelectrons decrease due to deceleration effect of the ionic Coulomb potential. Hence the hole shrinks and finally disappears at *t* = ∞. Meanwhile, the ring shrinks along with the hole and becomes a central peak, as indicated by the black curve in Fig. 7(b)) in the PMD eventually. Therefore, as discussed above, the formation of the central peak structure (i.e. ZES) can be attributed to the attraction of the ionic Coulomb potential, especially its long range tail, on the outgoing photoelectrons with low momenta after the laser pulse ends.

To deepen the understanding of the essential role of the long-range feature of Coulomb potential in the formation of the ZES, calculation with a truncated model potential is performed and the laser parameters are identical to those in Fig. 7(b). The results are shown in Fig. 7(c,d). This model potential is exactly identical to the Coulomb potential for *r* < 200 a.u. and is ramped off within 50 a.u. smoothly for 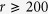 a.u. As shown in Fig. 7(c), a hole appears near zero momentum in the PMD at *t* = ∞. Accordingly, the LMD with integration over the transverse momentum interval of (0, 0.02 a.u.) at *t* = ∞ shows a dip at *p*_*z*_ = 0 (see black solid line in Fig. 7(d)), in contrast to a central peak with the Coulomb potential (see red dashed line in Fig. 7(d)). This can be understood by that, in the case of the truncated potential, most photoelectrons appear beyond the potential range at *t*_1_ and will not be decelerated after the laser pulse ends, resulting in disappearance of the ZES and therefore a hole in the final PMD. For *t* < *t*_1_, the photoelectron dynamics will be mainly determined by the laser field because it is compelling to the Coulomb potential for most of time. Consequently, the photoelectron momentum and spatial distributions at *t*_1_ will be very similar for the Coulomb and truncated potential. We indicate the edge of the truncated potential in Fig. 5(b,c) with dashed straight lines. It can be seen that most of photoelectrons, which will contribute to the ZES for the case of Coulomb potential, appear beyond the range of the truncated potential at *t*_1_. These photoelectrons can no longer feel any potential after *t*_1_, resulting in the absence of the ZES for the truncated potential. Therefore, the above analysis reveals that the long-range feature of Coulomb potential is indispensable for the formation of the ZES.

## Conclusion

To conclude, the photoelectron momentum distributions of noble gas atomic Xe in intense midinfrared laser fields are investigated both experimentally and theoretically. The PMDs and the LMDs with integration of the transverse momentum restricted to a very small range (i.e., 0–0.02 a.u.) show a zero-energy peak structure irrespective of laser intensity and wavelength. Moreover, its relative amplitude in the spectra varies with laser wavelength and intensity. A semiclassical simulation well reproduces these experimental observations qualitatively. Further analysis shows that these features in the momentum distributions can be attributed to the effect of the ionic Coulomb potential, especially its long range tail. Right after the laser pulse ends, the photoelectrons are still subject to the strong influence of the long range ionic Coulomb potential. Although their distances from the ionic core can be as large as 500 a.u., their momenta can be significantly altered by the ionic Coulomb potential. Moreover, the effect of the Coulomb potential on the final photoelectron momentum strongly depends on the spatial and momentum distributions of the photoelectrons right after the end of the pulse, while the latter depend on the laser parameters. Therefore, the relative amplitude of the ZES changes with laser wavelength and intensity. Our work shows that the Coulomb potential, especially its long-range part, plays an important role in determining the photoelectron dynamics. It is commonly accepted that in this very low energy region, since the De Broglie wavelength of the electron is very large, inherent quantum effects, such as quantum interference and diffraction, should play an important role. Nevertheless, our work shows that, surprisingly, the dynamics of very low-energy electron can be well described by an intrinsic classical picture, raising a challenge for relevant quantum approaches in the future.

## Methods

### Experimental technique

In our experiments, a commercial Ti: Sapphire femtosecond laser system (Legend, Coherent, Inc.) is implemented to generate femtosecond laser pulses with a repetition rate of 1 kHz, a pulse duration of 35 fs, and a center wavelength of 800 nm. This laser beam is applied to pump a commercial OPA laser system (TOPAS-C, Light Conversion, Inc.) to generate laser pulses with wavelengths varied from 1100 nm to 2400 nm with pulse duration of roughly 35 fs for signal and 55 fs for idler. To measure the pulse duration, the laser beam is frequency-doubled and directed into a commercial frequency-resolved optical grating (FROG) apparatus (Swamp Optics LLC. Grenouille Model 8–9 USB). The pulse shape is measured to be a near Gaussian distribution in both temporal and spatial domain. Since there are many cycles in one pulse, no CEP effect is expected in our experimental data. Therefore, we do not take any actions to actively phase stabilize the laser pulses. The maximum energy of the OPA laser beam varies from 1 mJ for 1300 nm, 750 *μ*J for 1800 nm, to 430 *μ*J for 2400 nm. The laser intensities are calibrated with a procedure utilizing photoelectron (photoion) momentum distribution in circularly polarized laser field^36^,^37^. The momentum distributions of photoelectrons and photoions are measured with a newly built Cold Target Recoil Ion Momentum Spectroscopy (COLTRIMS)^27^,^28^. Before directed into COLTRIMS, the laser pulse energy is precisely controlled with a combination of a broadband achromatic *λ*/2 plate and a broadband thin film polarizer. The photoelectrons and photoions created in the laser-supersonic beam interaction area are accelerated by a uniform weak electric field (2.3 V/cm) towards Microchannel Plates (MCP) detectors equipped with delay line anodes (HEX75 and DLD80, RoentDek Handels GmbH, for electron and ion respectively). The detectors can resolve their impact positions and time-of-flights, from which the 3 dimensional momenta of both fragments can be retrieved. A pair of Helmholtz coils generate a weak uniform magnetic field (2.3 Gauss) to confine the electron movement perpendicular to the electric field. The earth magnetic field is compensated with two pairs of auxiliary coils. A varied pinhole, which can be tuned with four micrometer drivers outside the vacuum chamber, is employed to reduce the cross section of supersonic beam and therefore the production rate of photofragments when necessary. During the experiments, each set of data consists of around 3.0 × 10^6^ ionization events.

### Simulation procedure

In our calculation, the laser electric field has a constant amplitude for the first 10 cycles and is ramped off within the next 3 cycles smoothly with a function of Cos square. The bound electron is firstly tunnel ionized randomly within the first 10 cycles. The ionization is described by quantum tunneling ionization theory^38^. Briefly, an electron with energy *K* = *I*_*p*_/4 tunnels through a one-dimensional effective potential *U*(*η*) = −1/4*η* − 1/8*η*^2^ − *Fη*/8, where *F* is the external field and *I*_*p*_ the ionization potential. The initial position (i.e., tunnel exit point *η*_0_) of the electron can be solved by *U*(*η*) = *K*, and the initial velocities are set to be *v*_*z*_ = 0, *v*_*x*_ = *v*_*per* _*cos θ*, *v*_*y*_ = *v *_*per*_ sin *θ*, where *v*_*per*_ is the transverse velocity and *θ* is the angle between *v*_*per*_ and *x* axis. The weight of each trajectory is evaluated by ADK formula 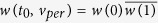
34 with


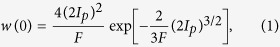



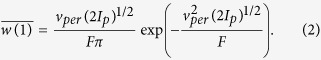


The subsequent evolution of the ionized electron in the combined Coulomb potential and the time-dependent intense laser field is governed by Newton’s equation of motion (in atomic units, *e* = *m* = *ħ* = 1):


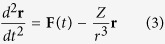


where 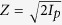 is the effective nuclear charge. The Newton’s equation of motion has been resolved by employing the standard Runge-Kutta algorithm. At the end of the laser pulse, the single ionization events are identified by energy criterion.

After the laser pulse ends, the momentum of electron at the detector (*r* → ∞) can be obtained analytically. Actually, the movement of an electron in the Coulomb field is within one plane. We can choose an appropriate coordinate system so that the movement is in the *y*-*z* plane. Then the trajectory is determined by the hyperbolic function


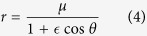


where 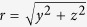, 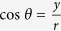, 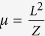, with *L* the angular momentum and *Z* the effective nuclear charge, 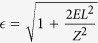, with *E* the total energy. Note that *E* and *L* are conserved here. The direction of the final momentum at *r* → ∞ is determined by the directrix of Eq. (4), so the final momentum can be expressed as 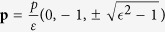, where 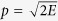. After the laser pulse ends, the energy *E* and angular momentum *L* are determined and the final momentum **p** in the chosen coordinate system can be obtained.

Next we need to perform a rotation of the coordinate system from (*x*, *y*, *z*) to the laboratory coordinate system (*x*_*l*_, *y*_*l*_, *z*_*l*_) to get the final momentum **p**_*l*_ = **R**(*α*, *β*, *γ*) **p** where **R** is the rotation matrix. The three Euler angles of the rotation matrix can be solved by the equation


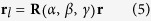


where both **r** and **r**_*l*_ are known: **r**_*l*_ is the coordinate of the electron just after the laser pulse ends, and the coordinate **r** in the chosen coordinate system can be obtained by Eq. (4), with 
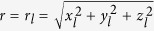
.

In the calculation, 7.2 × 10^7^ electron trajectories have been employed to assure the statistics. The results have been tested for numerical convergence by increasing the number of trajectories.

## Additional Information

**How to cite this article**: Quan, W. *et al.* Long-Range Coulomb Effect in Intense Laser-Driven Photoelectron Dynamics. *Sci. Rep.* **6**, 27108; doi: 10.1038/srep27108 (2016).

## Figures and Tables

**Figure 1:**
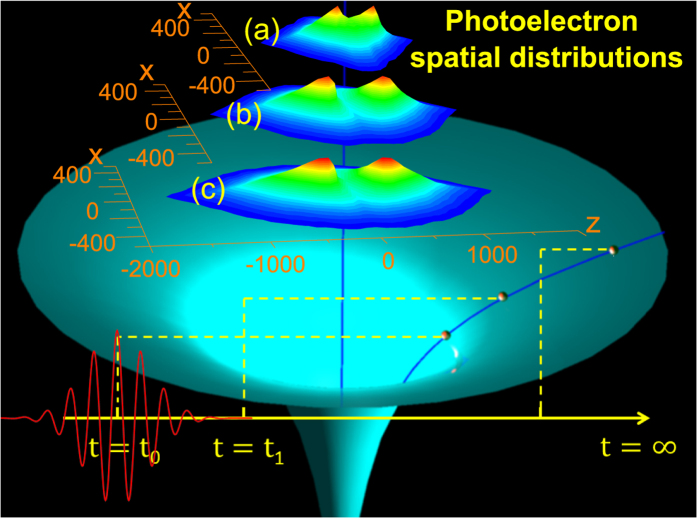
Calculated spatial distributions of photoelectrons in the plane of (*x*, *z*) right after the laser pulse ends (i.e., at *t* = *t*_1_). The laser polarization direction is in z axis. The laser wavelength is 1300 nm for (a) and 2400 nm for (b) and (c), while the laser intensity is 2.0 × 10^13^ W/cm^2^ for (a) and (b) and 3.0 × 10^13^ W/cm^2^ for (c), respectively. Under the spatial distributions is a schematic graph to demonstrate the influence of the long-range ionic Coulomb potential. When the electron is tunnel ionized, i.e., at *t* = *t*_0_, it appears at the tunnel exit, which is close to the ionic core. Right after the laser pulse vanishes at *t* = *t*_1_, the electron has been moved to some distance. After that and until the electron goes to infinity, it still experiences the Coulomb potential and the photoelectron momentum distributions will be altered by the long-range tail of ionic Coulomb potential.
